# Long non-coding RNA FAM87A is associated with overall survival and promotes cell migration and invasion in gastric cancer

**DOI:** 10.3389/fonc.2024.1448502

**Published:** 2024-09-25

**Authors:** Xue Jiang, Xiaobin Wu, Manjiao Lu, Wenna Fan, Jing Song, Fangzhou Song

**Affiliations:** ^1^ Molecular medicine and cancer research center, the basic school of Chongqing Medical University, Chongqing, China; ^2^ School of Smart Healthcare Industry, Chongqing City Management College, Chongqing, China; ^3^ Chongqing Health Center for Women and Children, Women and Children's Hospital of Chongqing Medical University, Chongqing, China

**Keywords:** FAM87A, gastric cancer, overall survival, ceRNA, invasion and metastasis, tumor microenvironment

## Abstract

**Background:**

The role of long non-coding RNAs (lncRNAs) in the invasion and metastasis of gastric cancer remains largely unclear.

**Methods:**

Integrating transcriptome data from The Cancer Genome Atlas (TCGA) and Gene Expression Omnibus (GEO) databases, differentially expressed genes were identified in gastric cancer. Using the Catalogue of Somatic Mutations in Cancer (COSMIC) database-curated gene set, lncRNAs associated with invasion and metastasis were identified. The Cox analyses were performed to identify prognostic lncRNAs. The competing endogenous RNA (ceRNA) regulation network was constructed to identify hub lncRNAs in gastric cancer. Functional and pathway analyses were used to investigate the function of identified lncRNAs. RT-qPCR and Transwell assays were used to investigate the expression in gastric cancer tissues and functions in gastric cancer cell lines.

**Results:**

Based on GEO and TCGA databases, 111 differentially expressed lncRNAs were identified between gastric cancer and normal samples. A total of 43 lncRNAs were significantly correlated with hallmark genes of cancer invasion and metastasis. Among them, as a hub lncRNA in the invasion-related ceRNA regulation network, FAM87A showed potential regulation on MAPK signaling and transforming growth factor (TGF) signaling cascade, such as TGFB2, TGFBR1, and TGFBR2. Furthermore, FAM87A also showed a significant correlation with cell adhesion molecules, such as Integrin alpha 6 (ITGA6) and Contactin-1 (CNTN1). RT-qPCR experiments showed that FAM87A expression was upregulated in gastric cancer tissues compared to normal samples (n = 30). Transwell assays showed that FAM87A knockdown inhibited the migration and invasion abilities of gastric cancer cells *in vitro*. Notably, clinical data analysis showed that lncRNA FAM87A could be an independent factor for the overall survival of patients with gastric cancer.

**Conclusion:**

LncRNA FAM87A may play a pivotal role in regulating migration and invasion of gastric cancer cells. FAM87A could be a potential biomarker for the overall survival of patients with gastric cancer.

## Introduction

Gastric cancer is the fifth most common cancer in the world. Gastric cancer was estimated as responsible for over 780,000 deaths worldwide in 2018, making it the third most lethal cancer type (1). The signs and symptoms associated with gastric cancer, if present, may include weight loss, nausea, dyspepsia, and epigastric pain (2). However, these symptoms may not occur until late in disease progression because they are vague and non-specific. Gastric cancer is diagnosed by endoscopic examination during which the tumor localization within the stomach and its macroscopic type are determined (3). More than half of the gastric cancer patients in the United States have regional or distant metastases at the time of diagnosis (4). Thus, the identification of key molecules associated with the invasion and metastasis of gastric cancer is critical for the development of new targeted drugs.

To date, studies on gastric cancer have primarily focused on the dysregulation of protein-coding genes to identify oncogenes and tumor suppressors for potential therapeutic targets. Long non-coding RNAs (lncRNAs) are RNA transcripts longer than  200 nt without coding potential (5). They do not necessarily have coding potential, but some lncRNAs have coding potential. They have been implicated in promoting invasion and metastasis in gastric cancer (6). Numerous investigations have demonstrated the regulatory role of lncRNAs in gene expression through various mechanisms, such as transcriptional and posttranscriptional processes (7). For instance, the lncRNA AK023391 has been shown to enhance invasion in gastric cancer by activating the PI3K/Akt signaling pathway (8). Nonetheless, the precise biological functions of lncRNAs in controlling the progression of gastric cancer remain poorly understood. Therefore, gaining a deeper comprehension of how lncRNAs contribute to the invasiveness and metastasis of gastric cancer will enhance our knowledge of the molecular mechanisms involved and potentially lead to advancements in targeted therapy for gastric cancer.

MicroRNAs (miRNAs) have the ability to induce gene silencing through mRNA binding, whereas lncRNAs can control gene expression through competitive binding with microRNA (9). Referred to as the competing endogenous RNA (ceRNA) hypothesis, substantial proof suggests that the ceRNA mechanism is prevalent in the progression of gastric cancer and holds significant importance (9). In this research, we analyzed variations in lncRNAs, miRNAs, and mRNAs among samples from gastric cancer and control groups to establish ceRNA networks for detecting pivotal lncRNA regulators. We conducted univariate and multivariate Cox regression tests to pinpoint lncRNAs linked to invasion and metastasis that exhibit strong predictive capability for the prognosis of patients with gastric cancer. Additionally, we cross-referenced several separate datasets to confirm the prognostic accuracy of these crucial lncRNAs.

## Materials and methods

### Clinical samples of gastric cancer

Fifteen gastric cancer tissues and paired healthy gastric tissues were collected from the First Affiliated Hospital of Chongqing Medical University (Chongqing, China). The solid tumor tissues of gastric cancer patients were obtained and immediately stored in liquid nitrogen after surgery. The informed consent of the patients was obtained. The ethics committee of Chongqing Medical University approved the use of patient tissues.

### Transcriptome data and preprocessing

The transcriptome data of stomach adenocarcinoma (STAD) samples and the corresponding clinical information of patients were downloaded from The Cancer Genome Atlas (TCGA; n = 407) and Gene Expression Omnibus [GEO; accession numbers are GSE66229 (n = 400) (10) and GSE64951 (n = 94) (11)]. To make the analysis more robust, three datasets were selected for analysis of differentially expressed lncRNAs in gastric cancer. First, the TCGA-STAD dataset is a good dataset with detailed clinical information and patient follow-up information. Second, as the TCGA-STAD expression dataset was based on RNA-seq technology, two expression datasets (GSE66229 and GSE64951) were further selected based on microarray technology to make the results more robust. The two GEO datasets have a higher sample size and the same platform GPL570, which is widely used in microarray-based studies and could detect more than 50,000 transcripts. This high detection rate of transcripts could better match the genes in RNA-seq technology (TCGA-STAD expression dataset). The level 3 HT-Seq count expression data of STAD were downloaded from the GDC data portal (https://portal.gdc.cancer.gov/, last accessed: July 5, 2020). The raw “.CEL” files of microarray data (Affymetrix Human Genome U133 Plus 2.0 array platform, GPL570) from the GEO database were downloaded and preprocessed using the affy package in the R software. The background correction and quantile normalization of samples in each microarray dataset were performed.

### Differential gene expression analysis

For the RNA-seq count data, the differential expression analyses were performed based on the DESeq2 package (12) in the R software. For the microarray data, the differential expression analyses were performed based on the limma package in the R software. The genes with Benjamini–Hochberg (BH)-adjusted p-value <0.05 were considered differentially expressed. The biotypes (protein-coding gene, miRNA gene, and lncRNA gene) of differentially expressed genes in each dataset were annotated based on the Ensembl release 90. For every two datasets, only genes that were detected in both sets but did not have a BH-adjusted p < 0.05 were filtered.

### Identifying genes associated with invasion and metastasis

First, the univariate Cox regression analysis based on the differentially expressed lncRNAs was performed to screen prognostic lncRNA genes. Then, the key lncRNA genes associated with invasion and metastasis were identified based on expression correlation analysis. Specifically, a 154-gene signature of invasion and metastasis in cancer was collected. This gene signature was extracted from the gene list of cancer census genes of Catalogue of Somatic Mutations in Cancer (COSMIC) release v92 (https://cancer.sanger.ac.uk/cosmic, last accessed: August 27, 2020) (13). The expression correlations between prognostic lncRNAs and cancer census genes were performed using Pearson’s correlation analysis. Gene pairs with Benjamini–Hochberg-adjusted p < 0.05 and Pearson’s coefficient |r| > 0.35 were considered significant.

### CeRNA network construction and hub gene identification

The ceRNA network was constructed using the predicted lncRNA–miRNA regulations and miRNA–mRNA regulations based on the miRcode database (v11) (14) and miRdb database (v6.0) (15), respectively. The commonly differentially expressed lncRNAs, miRNAs, and mRNAs were used as input to perform the analysis. The constructed ceRNA regulation networks were visualized using the Cytoscape software (version 3.7.2) “NetworkAnalyzer” application (version 2.7, September 2010) (16). To identify the hub lncRNA genes in the ceRNA network, multivariate Cox regression analysis was further performed in the TCGA cohort (training) and GSE66229 cohort (validation). The survival package (v3.3.1) was used to perform the patient survival analyses. The “Coxph” function was used for univariate and multivariate Cox model construction. The median expression of the lncRNA gene was used as a cut-off to divide the patients into high and low groups. The clinical parameters were also analyzed (as covariates) in the multivariate models.

### Functions and signaling pathways associated with the hub genes

The protein-coding genes in the subnetwork of the identified hub lncRNA were extracted and used to predict the functions and pathways of the hub lncRNA. Gene Ontology (GO) function and Kyoto Encyclopedia of Genes and Genomes (KEGG) pathway enrichment analyses were performed based on the clusterProfiler (v4.6.2) R package. GO terms and pathways with Benjamini–Hochberg-adjusted p-value <0.25 were considered significant. Correlation analysis between FAM87A and 44 genes in the FAM87A subnetwork was conducted using Spearman’s correlation analysis. Benjamini–Hochberg-adjusted p-value <0.05 was used as a threshold.

### Tumor microenvironment and immune cell infiltration analysis

In our study, correlation analyses utilizing Pearson’s method were conducted to examine the relationship between the expression levels of FAM87A and immune cell marker genes (17). Estimation scores for tumor infiltration of individual cell types in each sample were determined through various methods including CIBERSORT (18), EPIC (19), MCP-counter (20), quanTIseq (21), and TIMER (22). The impact of FAM87A on tumor infiltration in gastric cancer was investigated through Pearson’s correlation analyses of cell types. Significant gene–cell pairs were identified based on a threshold of p-values <0.05.

### Cell culture

Gastric cancer cell lines MKN-45 and HGC-27 were purchased from the American Type Culture Collection. Cells were cultured in a medium, followed by 10% fetal bovine serum (Shanghai ExCell Biology, Inc., Shanghai, China) supplemented with 100 U/mL penicillin and 100 μg/mL streptomycin at 37°C in a humidified atmosphere containing 5% CO_2_.

### Transfection

MKN-45 and HGC-27 cell lines were used to investigate the function of FAM87A in gastric cancer cells. FAM87A small interfering (si)RNAs and negative control (NC) were purchased from Shanghai GenePharma Co., Ltd. (Shanghai, China). A serum-free medium was used for transfection. After transfection for 6 h, cells were cultured in complete medium at 37°C for 48 h. GP-transfect-Mate (Shanghai GenePharma Co., Ltd.) was used for transfection. FAM87A siRNA-1, 5′-UUUACAGUCUCCCAGAUGGTT-3′; FAM87A siRNA-2, 5′-UCUAAAGGAUGCAUAGUGCTT-3′; and FAM87A siRNA-3, 5′-AUUUCGUUCUCUCCAUGCCTT-3′.

### Transwell assay

Cells were suspended in 200 µL serum-free medium and plated in the upper chambers of a Transwell plate. In the lower chambers, 600 µL of medium with 20% fetal bovine serum was used. After 36-h incubation, cells were washed twice with phosphate-buffered saline (PBS), fixed with methanol for 30 min, then stained with 0.1% crystal violet (Beyotime Biotechnology, Shanghai, China) for 20 min, and washed three times with PBS after gently wiping the inside surface of unmigrated cells. For Transwell invasion assays, Matrigel was applied to the upper chamber. Other steps for the invasion assay were the same as those for the migration assay. Three fields were selected randomly for counting and statistical analysis.

### Reverse transcription-quantitative PCR

The fresh frozen tissues were cut into pieces and treated by sonication. TRIzol^®^ reagent (Invitrogen, Carlsbad, CA, USA) was used to extract the total RNA of gastric cancer tissues and cells. PrimeScript™ RT Reagent Kit (Takara Bio, Inc., Mountain View, CA, USA) was used for reverse transcription. SYBR Premix Ex Taq™ (Takara Bio, Inc.) was used to perform qPCR experiments using thermocycling conditions, as follows: initial denaturation at 95°C for 30 sec, followed by 39 cycles at 95°C for 5 sec and 60°C for 30 sec. mRNA expressions of genes were quantified based on the 2^−ΔΔCq^ method (23) and normalized to the internal reference gene GAPDH. The primers of FAM87A were as follows: Forward 5′-AGTGGAAGAGAAGCATGGGC-3′; Reverse 5′-GTGAACAGATGCACGAACGG-3′. GAPDH

Forward: 5′-CCATGGGGAAGGTGAAGGTC-3′; Reverse: 5′-AGTGATGGCATGGACTGTGG-3′.

## Results

### Identification of differentially expressed lncRNAs, miRNAs, and protein-coding genes

**Figure 1:**
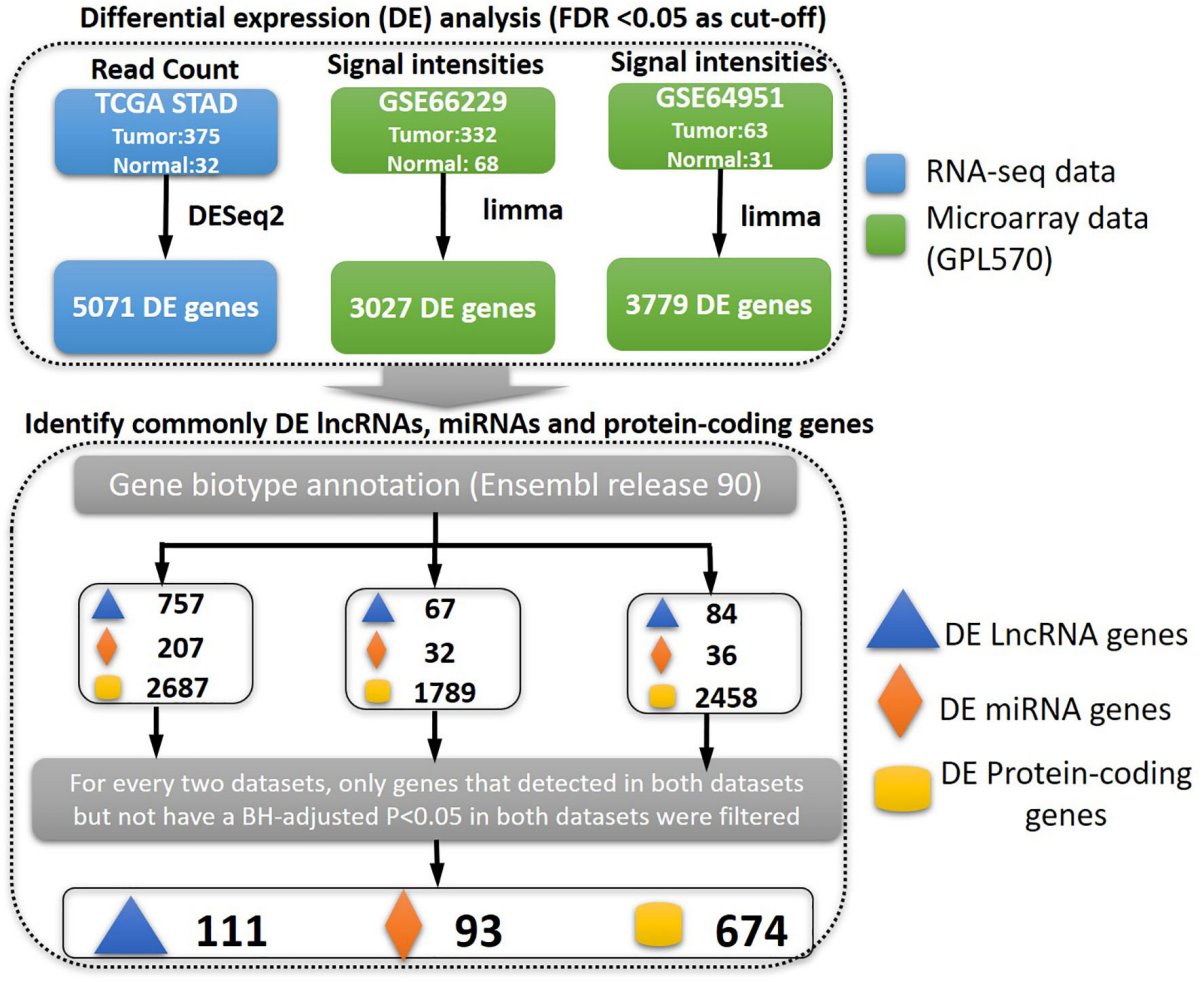
The pipeline for identifying differentially expressed genes. First, we identified the deregulated genes between gastric cancer and control samples based on the three types of genes interested (lncRNA, miRNA, or protein-coding genes). The gene biotypes were annotated based on Ensembl release 90. The commonly differentially expressed genes were finally identified.

In order to identify the genes that are abnormally regulated in STAD patients, an analysis of differential expression was conducted comparing samples from STAD patients to those from control subjects. To increase the reliability of the findings, three expression datasets were utilized using two distinct technologies, namely, RNA-seq and microarray. The TCGA dataset was obtained through RNA-seq, whereas the GSE66229 and GSE64951 datasets were acquired through microarray analyses. Subsequently, the biotypes of the differentially expressed genes, such as miRNA genes, lncRNA genes, and protein-coding genes, were annotated. The TCGA-STAD dataset was a mixed population (Asian, 81; Black or African American, 12; White, 223; other or unknown, 59). The GSE66229 and GSE64951 datasets comprised 100% Asian population. Genes not differentially expressed in both the GSE66229 and GSE64951 datasets were filtered, indicating that these differentially expressed genes (DEGs) may be robust in Asian patients. Then, genes that were identified as DEGs in the TCGA-STAD dataset and one of the Asian datasets (GSE66229 and GSE64951) were retained. In short, genes should have a consistent dysregulation status (BH-adjusted p < 0.05; fold change >1 for upregulation and fold change <1 for downregulation) in the three datasets, if detected. Ultimately, a total of 111 lncRNA genes, 93 miRNA genes, and 674 protein-coding genes were identified as DEGs (**Figure 1**).

### Identifying lncRNAs associated with invasion and metastasis and construction of ceRNA networks

**Figure 2:**
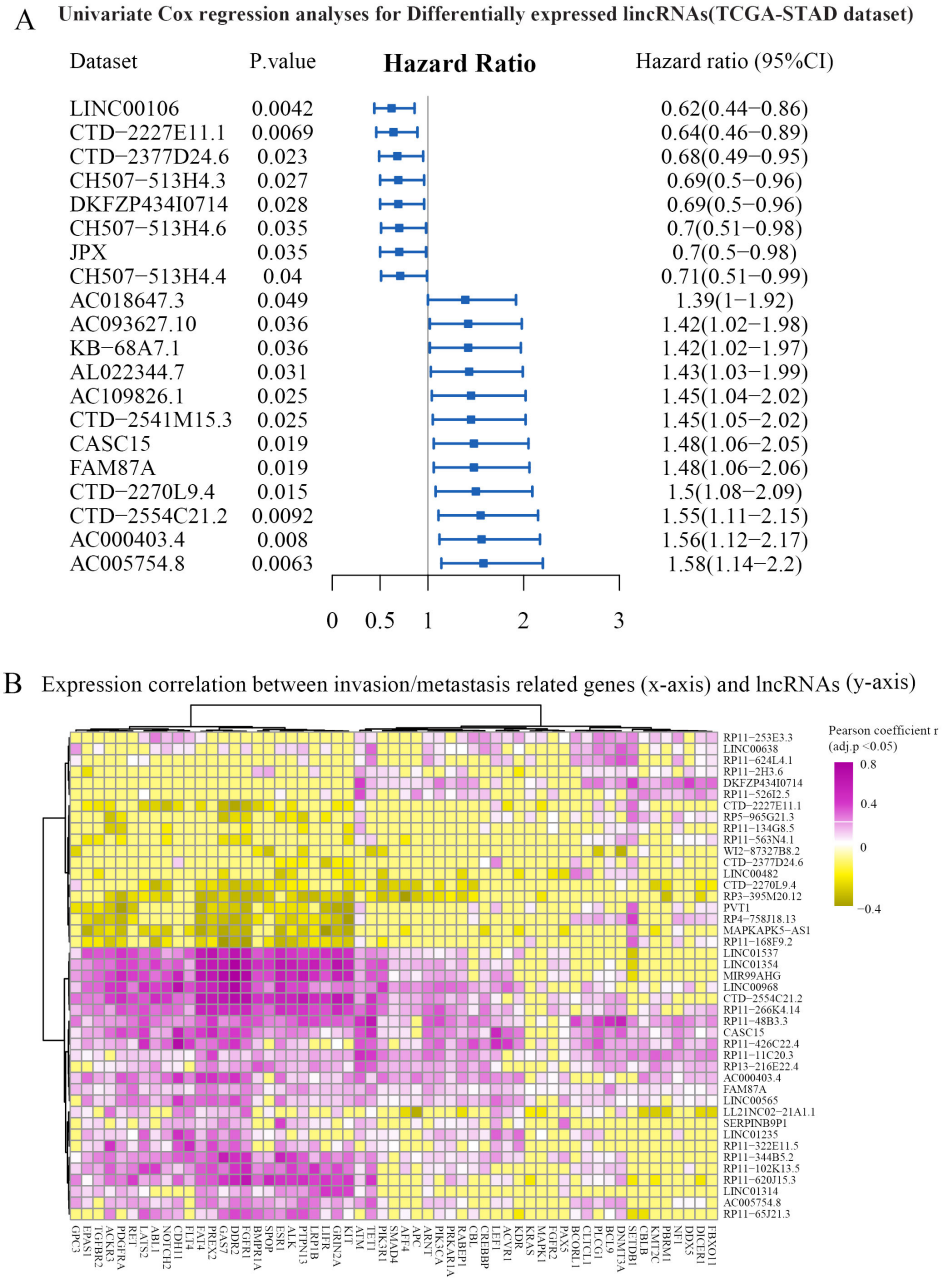
Identification of prognosis-related and invasion- and metastasis-related lncRNAs. **(A)** The univariate Cox regression analysis was performed based on differentially expressed lncRNAs. Representative results are shown. **(B)** Expression correlations between prognostic lncRNAs (y-axis) and invasion- and metastasis-related genes (x-axis). Hierarchical clustering (Euclidean distance with ward.D linkage) was used in the heatmap. The cells with Benjamini–Hochberg-adjusted p-value >0.05 were set to zero for better visualization. Genes significantly correlated with FAM87A are shown. CI, confidence interval.

**Figure 3:**
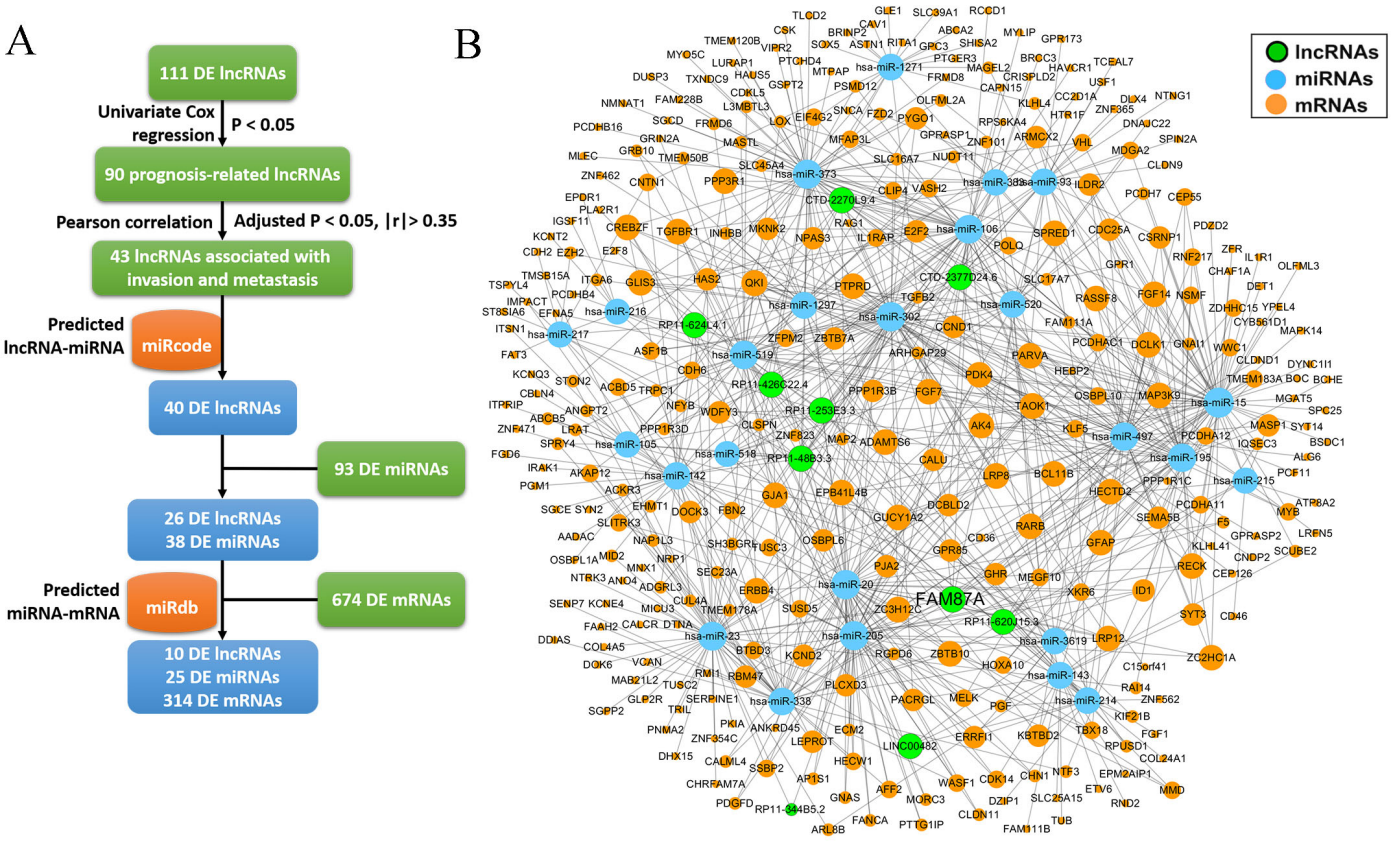
The construction of ceRNA network. **(A)** The pipeline to construct the ceRNA network based on miRcode and miRdb databases. **(B)** The ceRNA network (10 lncRNAs, 25 miRNAs, and 314 mRNAs) was visualized using Cytoscape software.

Invasion and metastasis are the hallmarks of stomach adenocarcinoma. To identify key lncRNAs associated with the progression of STAD, we collected a literature-curated 154-gene signature of “invasion and metastasis” from the COSMIC database (13). First, univariate Cox regression identified 90 lncRNA genes associated with patient overall survival of STAD (**Figure 2A**). Then, using Pearson’s correlation analysis, we further screened 43 lncRNAs significantly co-expressed with the 154 genes (**Figure 2B**). We used the TCGA-STAD dataset in the correlation analysis because of the higher detection rate (148/154 genes). Utilizing the miRcode and miRdb databases, we established the ceRNA networks by incorporating 10 lncRNAs, along with differentially expressed miRNAs and mRNAs (**Figure 3A**). We visualized the ceRNA networks using the Cytoscape software (**Figure 3B**).

### The hub lncRNA FAM87A is an independent biomarker for the overall survival of patients with STAD

**Figure 4:**
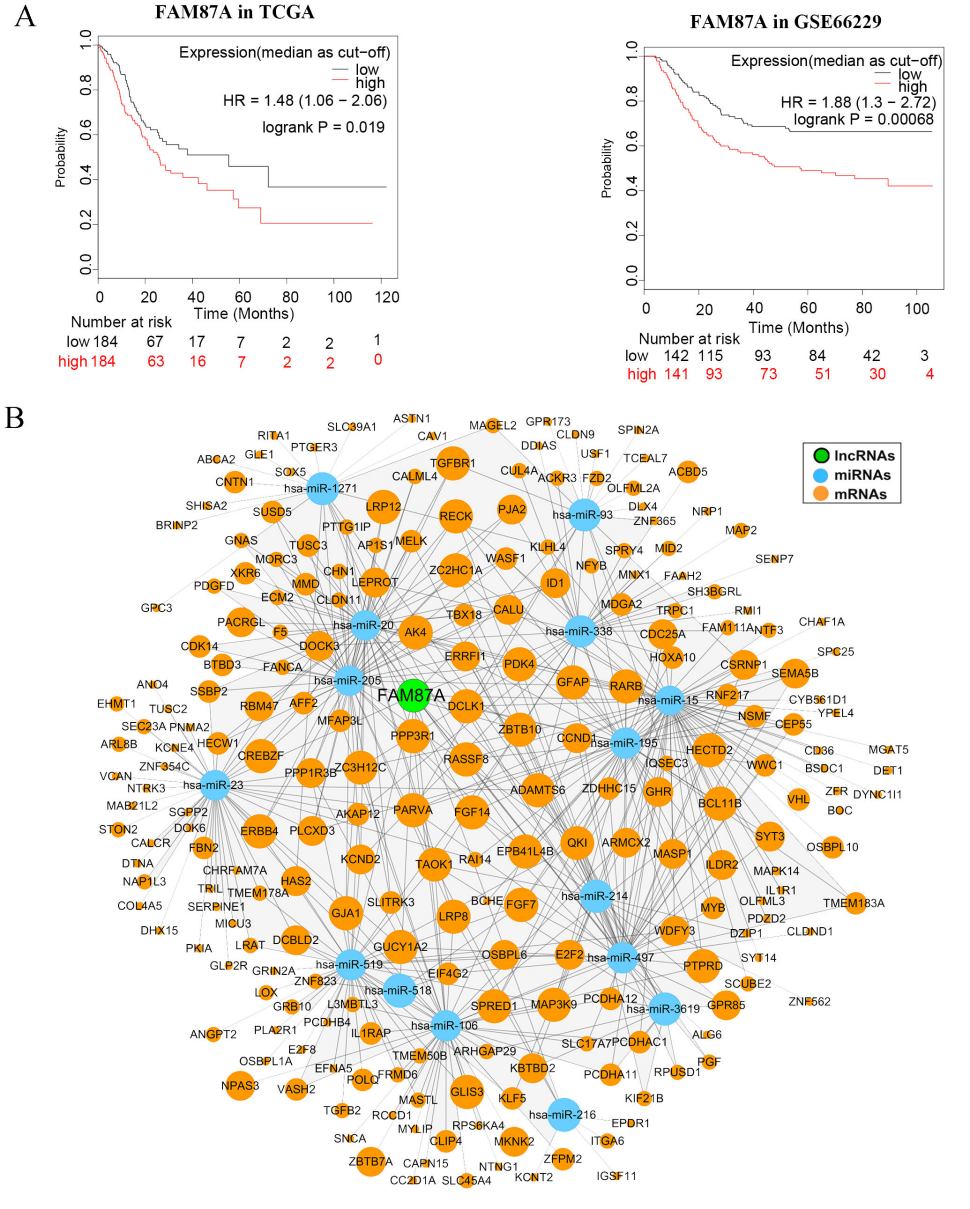
The Kaplan–Meier curves of FAM87A in gastric cancer and the FAM87A subnetwork. **(A)** The overall survival analysis of FAM87A in the TCGA dataset and GSE66229 dataset. **(B)** The sub-ceRNA network of FAM87A. HR, hazard ratio.

We further performed multivariate Cox regression analysis based on the expression of 10 lncRNAs in two independent cohorts (TCGA-STAD and GSE66229). We analyzed the clinical parameters (such as gender, age, and TNM stage) to test whether the lncRNA was independent of clinical covariates in the multivariate models. Results showed that FAM87A was an independent biomarker for the overall survival of patients with STAD (**Figure 4A**, log-rank p < 0.05). The multivariate models based on the training and validation cohorts are shown in **Table 1**. We further extracted the subnetwork of FAM87A (**Figure 4B**).

**Table 1:** The univariate and multivariate Cox regression models.

Variable	Univariate regression		Multivariate regression		Univariate regression		Multivariate regression	
	p-Value	Hazard ratio (95%CI)	p-Value	Hazard ratio (95%CI)	p-Value	Hazard ratio (95%CI)	p-Value	Hazard ratio (95%CI)
TCGA cohort (n = 375)					GSE66229 cohort (n = 332)			
FAM87A (high vs. low)	0.019	1.48 (1.0621~2.0632)	0.011	1.5731 (1.1108~2.2281)	6.82E−04	1.88 (1.3–2.72)	0.0016	1.8483 (1.2624~2.7061)
Age (ref, ≤67)					Age (ref, ≤63)			
>67	0.0113661	1.528 (1.1004~2.1217)	0.0002362	1.9639 (1.3705~2.8143)	0.2182	1.2552 (0.8742~1.8022)	9.00E−04	2.0184 (1.3346~3.0526)
Unknown	0.9945788	0 (0~Inf)	0.9947131	0 (0~Inf)	NA	NA	NA	NA
Gender (ref = female)								
Male	0.1676991	1.2814 (0.901~1.8224)	0.4415589	1.1535 (0.8018~1.6596)	0.6867	0.9249 (0.6331~1.3513)	0.3934	1.1911 (0.7972~1.7796)
Grade (ref = G1, highly differentiated)								
G2 (moderately)	0.4838408	1.658 (0.4026~6.8279)	0.4024898	1.8968 (0.4238~8.4897)	NA	NA	NA	NA
G3 (poorly)	0.2700981	2.2007 (0.5417~8.9408)	0.2479433	2.3798 (0.5467~10.359)	NA	NA	NA	NA
Unknown	0.2285287	2.8391 (0.5195~15.5173)	0.2620478	2.8728 (0.4544~18.1633)	NA	NA	NA	NA
pStage (ref = I)								
II	0.1934812	1.5453 (0.8019~2.9779)	0.8277389	0.8983 (0.3418~2.3608)	0.1667	2.3434 (0.7009~7.8353)	0.7628	1.2821 (0.2553~6.4372)
III	0.0093958	2.261 (1.2216~4.1846)	0.9604044	0.9676 (0.2633~3.5551)	0.0062	5.1535 (1.5915~16.6875)	0.5933	1.6274 (0.2725~9.7178)
IV	0.000117	3.9094 (1.9537~7.823)	0.3021443	2.103 (0.5124~8.6312)	0	14.078 (4.3914~45.1313)	0.2642	2.8495 (0.4534~17.9079)
Unknown	1.097E−05	10.64 (3.7085~30.5269)	0.0615994	14.967 (0.8768~255.4766)	NA	NA	NA	NA
pM (ref = M0, without distant metastasis)								
M1 (with distant metastasis)	0.0046735	2.225 (1.2784~3.8724)	0.5720206	1.2815 (0.5422~3.0288)	0	4.3291 (2.6805~6.9918)	0.0395	2.1129 (1.0366~4.3067)
Unknown	0.3164392	1.521 (0.6696~3.4547)	0.6402614	1.2516 (0.4883~3.2077)	NA	NA	NA	NA
pN (ref = N0, 0 regional lymph node metastasis)								
N1 (1–2)	0.0640359	1.5756 (0.9738~2.5494)	0.537825	1.2386 (0.627~2.4467)	0.2342	1.6335 (0.7278~3.6665)	0.482	1.5137 (0.4766~4.8076)
N2 (3–6)	0.0760192	1.6008 (0.9519~2.6919)	0.5603514	1.2875 (0.55~3.0137)	0.0024	3.4917 (1.555~7.8406)	0.1595	2.4955 (0.698~8.9212)
N3 (≥7)	7.967E−05	2.6228 (1.6245~4.2344)	0.1146611	1.9928 (0.8461~4.6935)	0	7.8976 (3.5087~17.7765)	0.0506	3.6877 (0.9968~13.6435)
Unknown	0.0289347	2.8976 (1.1155~7.5269)	0.1499151	2.4403 (0.7245~8.2198)	NA	NA	NA	NA
pT (ref = T1, tumor invades lamina propria, muscularis mucosae, or submucosa)					pT (ref = T2)			
T2 (tumor invades the muscularis propria)	0.0626392	6.6631 (0.9048~49.0683)	0.1212466	5.0738 (0.6504~39.58)	NA	NA	NA	NA
T3 (tumor invades the subserosal connective tissue)	0.024963	9.564 (1.3285~68.8541)	0.0954098	6.2907 (0.7243~54.6338)	0	2.4205 (1.6445~3.5626)	0.27	1.3406 (0.7963~2.257)
T4 (tumor invades the serosa or adjacent structures)	0.0261189	9.5193 (1.3072~69.3203)	0.1519606	4.9635 (0.5545~44.4307)	5.00E−04	2.8445 (1.5813~5.1169)	0.8181	0.9162 (0.4344~1.932)

The median expression of the gene was used as the cut-off to divide patients into high or low groups. NA represents no samples in that category or all samples belong to the same category. The American Joint Committee on Cancer (AJCC) staging system 8th edition was used.

CI, confidence interval; TCGA, The Cancer Genome Atlas.

### Functional and pathways analysis of FAM87A subnetwork

**Figure 5:**
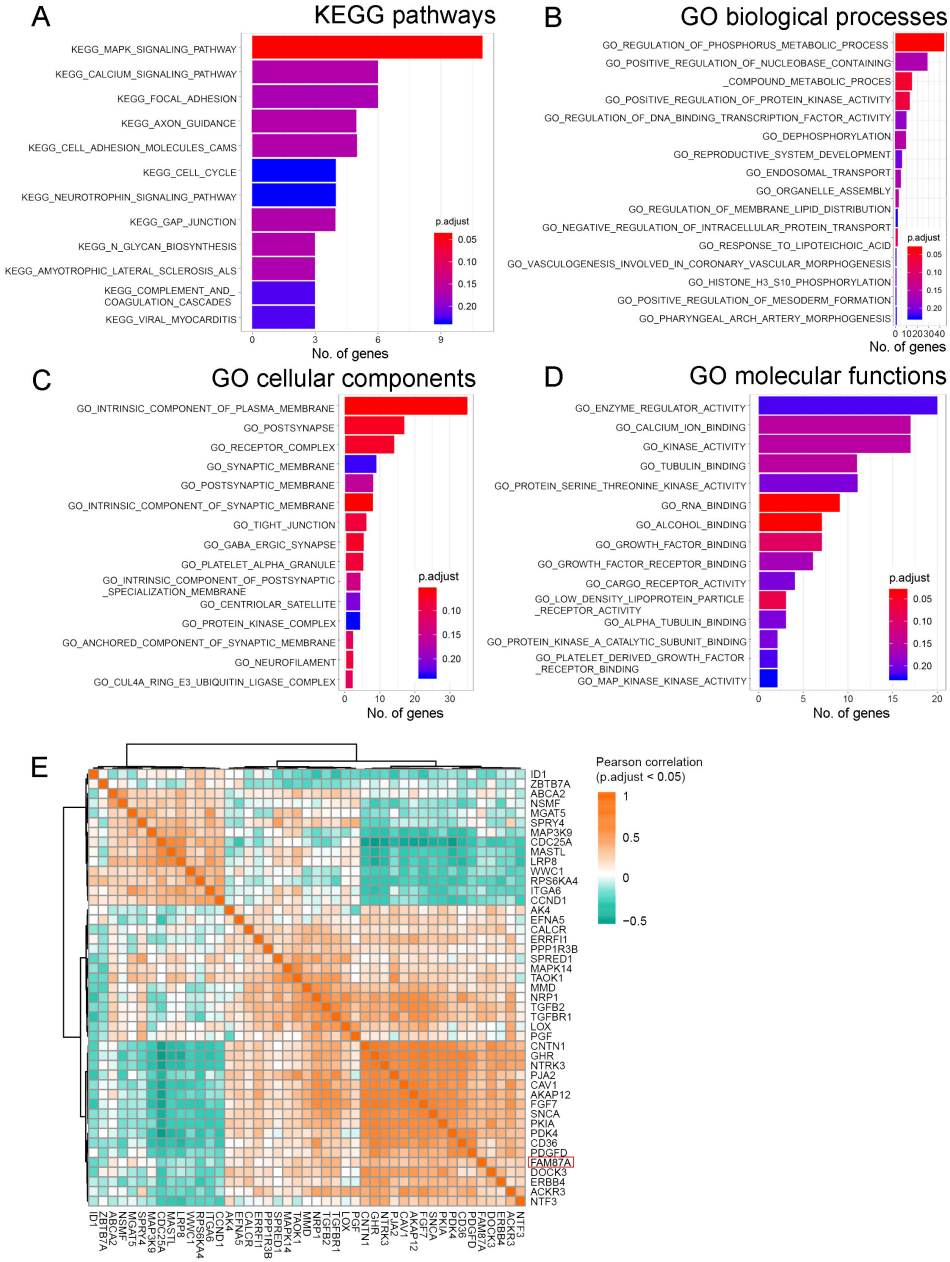
The functional and pathway enrichment analyses of FAM87A. **(A)** Kyoto Encyclopedia of Genes and Genomes (KEGG) pathways involved, based on the protein-coding genes in the FAM87A ceRNA network. **(B–D)** Gene Ontology (GO) functional terms involved in biological processes, cellular components, and molecular functions, respectively. **(E)** Pearson correlation analysis between FAM87A and genes involved in the regulation of phosphorus metabolic process.

We further investigated the function and pathways of the FAM87A subnetwork. We performed the GO functional and KEGG pathway enrichment analyses based on the protein-coding genes extracted from the FAM87A subnetwork. Results showed that FAM87A-associated genes (**Supplementary Table S1**) were mainly involved in the MAPK signaling pathway (**Figure 5A**, p.adjust < 0.05). We performed Gene Ontology enrichment analysis based on the genes in the subnetwork. The involved biological processes include the regulation of the phosphorus metabolic process and the regulation of protein kinase activity (**Figure 5B**, p.adjust < 0.05). The involved cellular components include plasma and synaptic membrane (**Figure 5C**, p.adjust < 0.05). The involved molecular functions include protein binding and receptor activity (**Figure 5D**, p.adjust < 0.2). Correlation analysis between FAM87A and the gene involved in the regulation of the phosphorus metabolic process indicated that FAM87A was significantly correlated with metabolic reprogramming-related genes, such as Caveolin-1 (CAV1; **Figure 5E**).

### The association between FAM87A and immune-infiltrating cells

**Figure 6:**
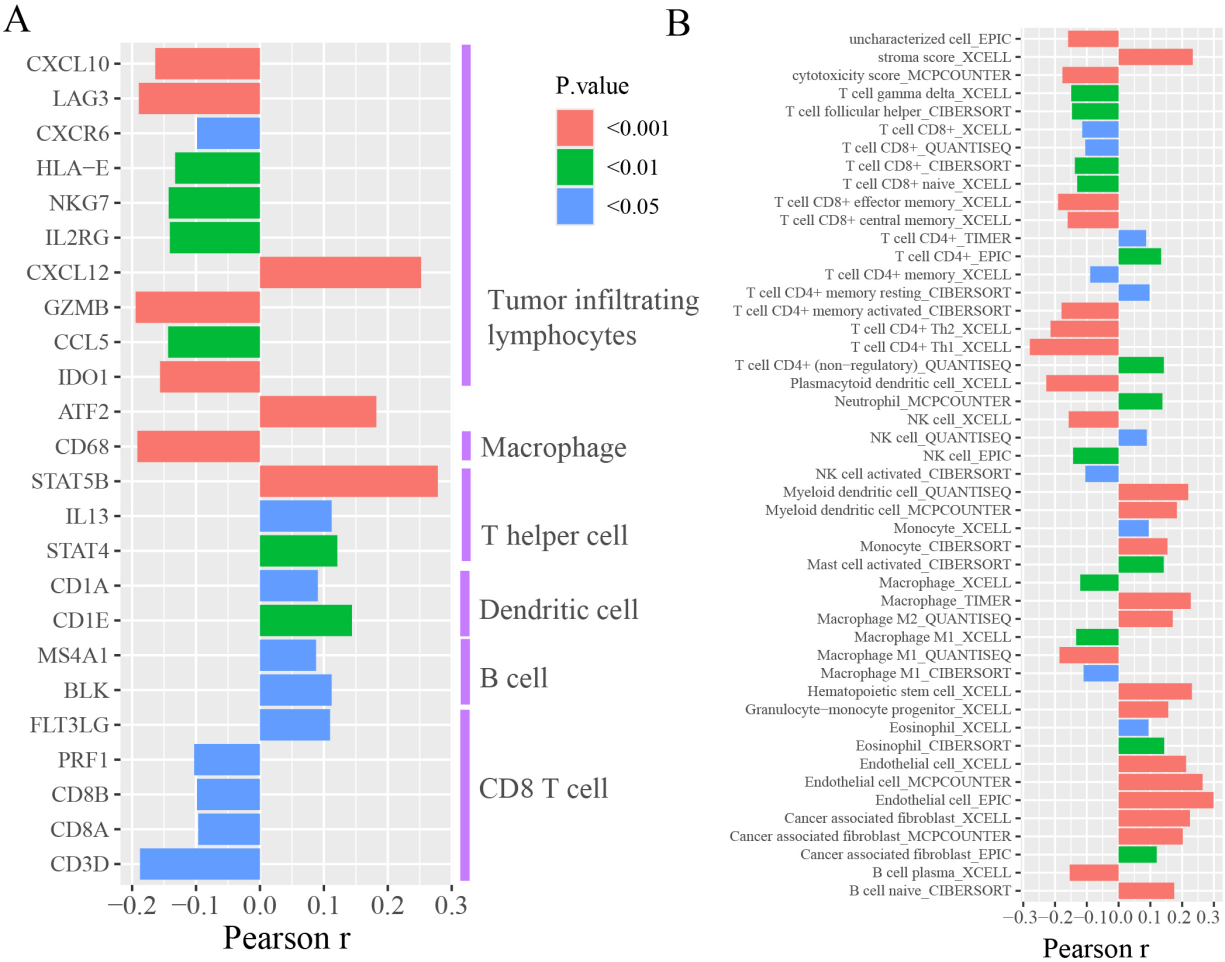
The tumor microenvironment analysis of FAM87A in gastric cancer. **(A)** The expression correlation between FAM87A and the marker genes of immune cells. **(B)** The correlation between single sample tumor-infiltrating estimation scores (estimated based on CIBERSORT, EPIC, MCP-counter, quanTIseq, xCell, and TIMER methods) and FAM87A expression in gastric cancer.

We further investigated the correlation between FAM87A and the immune microenvironment of tumors. We performed an expression correlation analysis between the biomarkers of immune cells and FAM87A using the TCGA-STAD dataset. Results showed that FAM87A was closely correlated with tumor-infiltrating lymphocytes (**Figure 6A**). Consequently, we further analyzed the cells infiltrating in association with FAM87A. Results indicated that FAM87A was primarily associated with macrophages, T cells, dendritic cells, and cancer-associated fibroblasts (**Figure 6B**, **Supplementary Table S2**).

### FAM87A downregulation inhibits migration and invasion of gastric cells *in vitro*

**Figure 7:**
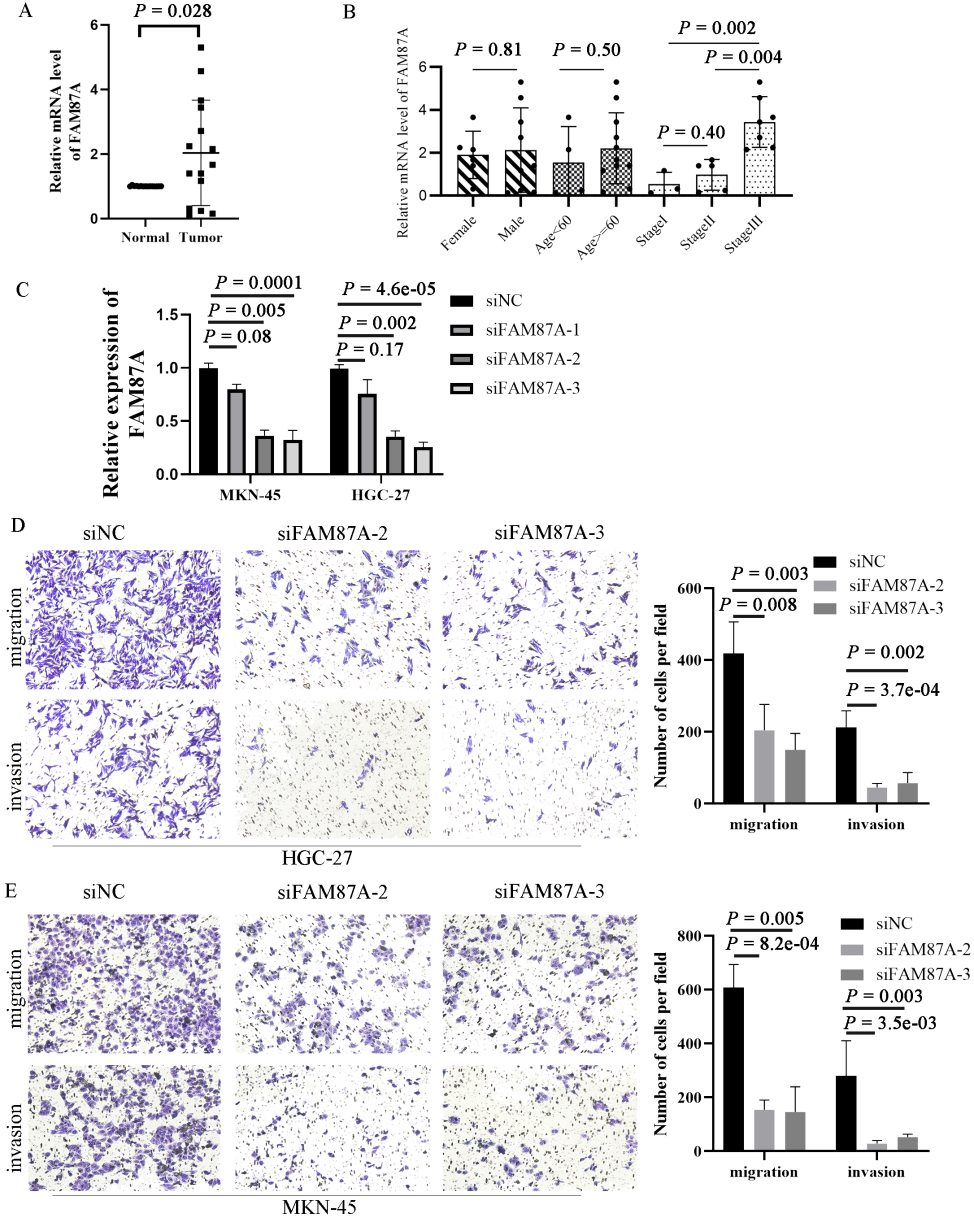
Expression validation and functional experiments of FAM87A in gastric cancer. **(A)** The expression of FAM87A in tissues of patients with gastric cancer (n = 30). **(B)** The relative mRNA expression of FAM87A in gastric cancer patients with different patient characteristics and clinical stages. The mRNA levels of FAM87A showed no significant difference between male patients and female patients, as well as between elderly patients and younger patients (median as cut-off). Although there is no significant difference in FAM87A expression between stage II and stage I patients, the expression of FAM87A was significantly increased in stage III patients. **(C)** Transfection efficacy of siRNAs of FAM87A. **(D, E)** Transwell assays of gastric cancer cells between FAM87A knockdown and negative control groups. Two-tailed Student’s t-test was used for statistical analysis.

To validate the expression of FAM87A in tissues of patients with gastric cancer, we collected 15 pairs of samples from the First Affiliated Hospital of Chongqing Medical University. The patient information is summarized in **Table 2**. Reverse transcription-quantitative PCR (RT-qPCR) results showed that the expression of FAM87A was upregulated in gastric cancer samples compared to healthy samples (p = 0.0278, **Figure 7A**). Furthermore, analysis of FAM87A expression based on clinical information showed that FAM87A expression was higher in stage III patients compared with either stage I or stage II patients. In contrast, there is no significant difference in FAM87A expression between men and women, so that between older patients (≥60 years) and younger patients (<60 years, **Figure 7B**). SiRNA infection of FAM87A showed that #2 and #3 siRNAs of FAM87A have a higher efficacy (**Figure 7C**). Then, we investigated the functional role of FAM87A in gastric cancer cells. Transwell assays showed that a greater number of gastric cancer cells migrated from the inside surface to the outside surface after silencing of FAM87A. Similarly, when we applied Matrigel in the upper chamber, there were still a higher number of gastric cancer cells invading the outside surface. The data indicated that FAM87A knockdown inhibited the migration and invasion abilities of gastric cancer cells, compared with the negative control (**Figures 7D, E**).

**Table 2:** Patient characteristics of 15 patients with gastric cancer.

Characteristics	No. of patients (percentage)
Gender	
Female	6 (40%)
Male	9 (60%)
Age	
<60	4 (26.7%)
≥60	11 (73.3%)
pT (tumor size)	
T1	3 (20%)
T2	3 (20%)
T3	6 (40%)
T4	3 (20%)
pN (lymph node metastasis)	
N0	4 (26.7%)
N1	5 (33.3%)
N2	5 (33.3%)
N3	1 (6.7%)
pM (distant metastasis)	
M0	15 (100%)
pStage	
I	3 (20%)
II	5 (33.3%)
III	7 (46.7%)

## Discussion

During this research project, various lncRNAs, miRNAs, and mRNAs were examined for differential expression in gastric cancer samples compared to control samples. Analysis using the univariate Cox method identified 90 lncRNAs that were associated with prognosis. Then, 43 invasion and metastasis-associated lncRNAs were identified in gastric cancer. The ceRNA network was constructed based on the 43 lncRNAs and differentially expressed miRNAs and mRNAs. Further network analysis and multivariate Cox analysis identified the core subnetwork and the hub lncRNA FAM87A. FAM87A could be an independent biomarker for the overall survival of patients with gastric cancer. Patients with higher expression of FAM87A have significantly poorer overall survival, which has been validated by an additional independent cohort.

FAM87A is relatively novel in cancer. Zhang et al. revealed that FAM87A was associated with the overall survival of patients with tongue squamous cell carcinoma (24). In this study, in fact, we designed the analysis pipeline to identify invasion- and metastasis-related lncRNAs. After differentially expressed lncRNAs in common among three expression datasets were identified, we showed that 43 lncRNAs were significantly correlated with hallmark genes of cancer invasion and metastasis. The hallmark gene set was curated by the COSMIC database (154 genes, release v92, https://cancer.sanger.ac.uk/cosmic, last accessed: August 27, 2020). Among the 43 lncRNAs, as a hub lncRNA in the invasion-related ceRNA regulation network, FAM87A showed potential regulation on transforming growth factor (TGF) signaling cascade, such as TGFB2, TGFBR1, TGFBR2, and TGFBR3/FLT4. These genes and TGF-beta signaling were previously demonstrated as crucial processes in cancer invasion and metastasis. Furthermore, FAM87A also showed a significant correlation with cell adhesion molecules, such as Integrin alpha 6 (ITGA6, **Figure 5E**) and Contactin-1 (CNTN1, **Figure 5E**). CNTN1 is a key downstream effector of VEGFR3/FLT4 (**Figure 5E**), and this axis was involved in the regulation of invasive capacity in different types of cancer cells. Upon these results and evidence, we hypothesized that FAM87A regulated cell migration and invasion of gastric cancer cells and performed the cell line experiments.

The metabolic reprogramming was closely associated with most stages of cancer progression, including invasion and metastasis (25). Cumulative studies have highlighted the application of metabolomics in gastric cancer research regarding different aspects (26). Based on the GO functional results, the involved biological processes of FAM87A were regulation of phosphorus metabolic process, regulation of nucleobase containing compound metabolic process, and regulation of protein kinase activity. Based on the correlation analysis; we found that FAM87A was associated with many metabolic reprogramming-related genes, such as CAV1, pyruvate dehydrogenase kinase 4 (PDK4), and transforming growth factor beta 2 (TGFB2). CAV1 interacts with insulin and IGF-1 receptors (IR/IGF-1R) to stimulate IR/IGF-1R signaling and enhance glucose uptake through AKT signaling activation and lactate output in prostate cancer (27). PDK4 is an energy metabolism-related protein. *N* ^6^-Methyladenosine (m^6^A) modification in 5′-UTR of PDK4 positively regulated the mRNA stability and translation of PDK4 to promote glycolysis in cancer cells (28). In contrast, TGFB2 was demonstrated to upregulate the lipogenesis regulator sterol regulatory element binding factor 1 and its downstream lipogenic enzymes via PI3K–AKT signaling in pancreatic ductal adenocarcinoma (29). This evidence, combined with our results, indicated that FAM87A may play a role in regulating metabolic reprogramming.

We also analyzed the association between FAM87A and immune-infiltrating cells. Multiple algorithms showed concordant results that FAM87A was mainly associated with T cells, dendritic cells, and cancer-associated fibroblasts, revealing that FAM87A may play a role in tumor infiltration and the tumor microenvironment. The absence of significant enrichment of immune-related pathways may be because of the small number of immune-related genes in the FAM87A subnetwork. However, we showed that FAM87A was significantly positively related to CD36 (**Figure 5E**), which is a critical protein in regulating tumor-associated macrophage and cytotoxic T cells in the tumor microenvironment. Tumor cells reprogram the metabolism and immunity of M2-polarized macrophages by releasing lipid-carrying vesicles and increasing CD36 to promote liver metastasis (30). Moreover, it was demonstrated that CD36 mediated the uptake of fatty acids by tumor-infiltrating CD8 T cells to induce lipid peroxidation and ferroptosis, resulting in reduced cytotoxic cytokine production and impaired antitumor ability (31). These data echo our results that FAM87A was negatively associated with cytotoxicity score and positively related to M2 macrophages (**Figure 6B**). Thus, FAM87A may be involved in the tumor microenvironment and associated with metastasis by regulating CD36. Taken together, FAM87A is a key lncRNA in the invasion and metastasis of gastric cancer.

## Conclusion

LncRNA FAM87A may play a pivotal role in regulating migration and invasion of gastric cancer cells. FAM87A could be a potential biomarker for the overall survival of patients with gastric cancer.

## Data Availability

The datasets presented in this study can be found in online repositories. The names of the repository/repositories and accession number(s) can be found in the article/**Supplementary Material**.
